# Two-State Co-Expression Network Analysis to Identify Genes Related to Salt Tolerance in Thai Rice

**DOI:** 10.3390/genes9120594

**Published:** 2018-11-29

**Authors:** Apichat Suratanee, Chidchanok Chokrathok, Panita Chutimanukul, Nopphawitchayaphong Khrueasan, Teerapong Buaboocha, Supachitra Chadchawan, Kitiporn Plaimas

**Affiliations:** 1 Department of Mathematics, Faculty of Applied Science, King Mongkut’s University of Technology North Bangkok, Bangkok 10800, Thailand; apichat.s@sci.kmutnb.ac.th; 2 Advanced Virtual and Intelligent Computing (AVIC) Center, Department of Mathematics and Computer Science, Faculty of Science, Chulalongkorn University, Bangkok 10330, Thailand; chorn.chid@gmail.com; 3 Department of Botany, Faculty of Science, Chulalongkorn University, Bangkok 10330, Thailand; priggerr@gmail.com (P.C.); sharesci@gmail.com (N.K.); supachitra.c@chula.ac.th (S.C.); 4 Department of Biochemistry, Faculty of Science, Chulalongkorn University, Bangkok 10330, Thailand; teerapong.b@chula.ac.th

**Keywords:** salt-tolerant genes, gene co-expression network, RNA sequencing, network analysis, ‘Khao Dawk Mali 105’, clustering coefficients, two-state networks, salt stress, rice genome

## Abstract

Khao Dawk Mali 105 (KDML105) rice is one of the most important crops of Thailand. It is a challenging task to identify the genes responding to salinity in KDML105 rice. The analysis of the gene co-expression network has been widely performed to prioritize significant genes, in order to select the key genes in a specific condition. In this work, we analyzed the two-state co-expression networks of KDML105 rice under salt-stress and normal grown conditions. The clustering coefficient was applied to both networks and exhibited significantly different structures between the salt-stress state network and the original (normal-grown) network. With higher clustering coefficients, the genes that responded to the salt stress formed a dense cluster. To prioritize and select the genes responding to the salinity, we investigated genes with small partners under normal conditions that were highly expressed and were co-working with many more partners under salt-stress conditions. The results showed that the genes responding to the abiotic stimulus and relating to the generation of the precursor metabolites and energy were the great candidates, as salt tolerant marker genes. In conclusion, in the case of the complexity of the environmental conditions, gaining more information in order to deal with the co-expression network provides better candidates for further analysis.

## 1. Introduction

Rice is one of the most important agricultural products of Thailand with the highest domestic consumption and exportation. Khao Dawk Mali 105 (KDML105) rice, known as jasmine rice, is considered a high-quality rice from Thailand. It is grown mainly in the northeastern region of Thailand, where the soil contains a high salinity. This poor soil quality affects plant growth and productivity. With the ability to grow in high salinity areas, the salinity tolerance score of KDML105 is lower than some other rice varieties, such as IR29, KKU-LLR-039 and Niewdam Gs. No. 88084 [1]. This information suggested that KDML105 rice should contain some important salt tolerant genes.

In order to identify the key gene(s) playing an important role in salt tolerance, the high-throughput technology of RNA sequencing (RNA-seq) has been applied. New transcripts, single nucleotide polymorphisms (SNP), and gene and splice variants could be identified using this technique [2,3]. In addition, this technology is widely used for the study of differential gene expressions during plant responses to various stressors [2,3,4,5,6,7,8]. For example, the study of Zhang and his coworkers [3] reported the transcriptome analysis of rice roots in response to potassium deficiency. Furthermore, beyond the gene expression level, the analysis of a gene co-expression network has become a broadly used technique for searching for important markers or genes that are in cooperation with other genes. However, this network analysis is quite rare and needs a more elaborate method to represent as much as information regarding the strength of the connections. Subsequently, various network-based analyses have been applied to the gene co-expression network [9,10,11,12,13,14,15,16,17]. Some of the simple and widely used local network measurements are the degree centrality (or hub centrality) [13,14,17,18,19] and clustering coefficient [17,20,21,22,23,24,25]. The study of Chen and his colleagues employed a co-expression network analysis to identify the hub genes associated with metastasis risk and prognosis in cancer cells [9]. Zhang and Horvath applied clustering coefficient on a gene co-expression network of cancer in humans, and found that the clustering coefficient could be used for distinguishing the difference between normal genes and cancer genes [17]. The method using the clustering coefficient gave better results, as compared to other methods using a simple measure of degree of connectivity [17]. The development of the clustering coefficients has been implemented for the RNA-seq dataset [15,16]. However, the constructed co-expression networks are typically performed as a binary graph by ignoring the strength of the connections between the genes. The relevance of genes can be derived from the correlations of the gene expression levels of all of the gene pairs in the network. However, calculating the correlations of all of the gene pairs in the network causes a high computational complexity in terms of the network analysis. Fortunately, the computations with greater data can presently be performed with high performance computing technologies. Thus, the analysis of a weighted network is possible, and is of interest for its application in the real biological network. Li and his coworkers used a weighted gene co-expression network analysis to identify the novel potential genes involved in the early metamorphosis process in sea cucumbers [11]. Jianqiang Li and his colleagues developed a weighted gene co-expression network analysis framework to analyze forty oral squamous cell carcinoma (OSCC) specimens as well as their matched non-tumorous epithelial counterparts [10].

For the network analysis, several topological features are widely employed to identify new target genes. Degree is one of the most commonly used features to identify a local connection. It represents the number of neighboring nodes in the network. The hub genes in the network are the genes with high degrees, which are mostly known to be important genes for the structure of the network and biological functions [13,14,26]. This means that the higher the number of neighbors for a considered gene, the more implied importance the gene has than others in term of maintaining the network structure as well as the organisms’ lives [13,14,26]. However, information regarding the connections among neighbors is still lacking. The clustering coefficient is another local density-connection measure that focuses on connections among neighbors, and it has been developed for various kinds of networks from a simple binary network to a weighted network, as well as a recent version for a signed weighted network, which mostly fulfils with any real-world networks [13,17,23,27,28]. Thus, the clustering coefficients have been used in many fields [20,21,22,23,24,28,29,30,31,32,33]. The clustering coefficient for a weighted network was applied to the financial and metabolic networks by using the geometric mean of the weights [28]. The application of the clustering coefficient was also done for a gene co-expression network of simulated data, a cancer microarray data set, and a yeast microarray data set [17]. The results show that, in a scale-free network, the weighted clustering coefficient was not inversely related to the connectivity, while the binary clustering coefficient was inversely related to connectivity [17].

In this work, we studied the whole transcriptome sequencing data of KDML105 under salt-stress and control situations. The complete signed gene co-expression network was constructed for both conditions, and the clustering coefficients were applied to the networks. The aim is to compare the different structures of the normal-state network and salinity-state network using a clustering coefficient and degree on the weighted co-expression network. Finally, potential target genes were selected and proposed for further study and experimental validation.

## 2. Materials and Methods

### 2.1. Plant Material Preparation

KDML105 rice at seedling and booting stages were used as the plant materials. In rice, the seedling stage is a part of the vegetative phase of rice growth, developing more leaves and seminal roots after the first root and shoot of the rice emerge following germination. The booting stage is the first step in the reproductive phase of rice. In this stage, a bulging of the leaf stem conceals the developing panicle. Rice during both seedling and booting stages is sensitive to the salt stress. The experiments were conducted using three biological replicates, each consisting of one plant. The KDML seeds were soaked in distilled water for five days in plastic cups until germination. The germinated seeds were transplanted into the plastic trays for the seedling samples, and the booting samples were transplanted into the plastic plots. All plants were grown in a Bangsai nutrient solution that contained 50 g/L of MgSO_4_, 80 g/L of KNO_3_, 12.5 g/L of NH_4_H_2_PO_4_, 8.5 g/L of KH_2_PO_4_, 0.4 g/L of Manganese chelate of ethylenediaminetetraacetic acid (Mn-EDTA), 0.8 g/L of micronutrient, 100 g/L of Ca(NO_3_)_2_, and 3 g/L of Ferric iron chelate of ethylenediaminetetraacetic acid (Fe-EDTA). For the seedling stage of the experiment, the plants were grown in soil, in the pot 3 × 3 × 7 cm^3^ in size for 18 days after germination. Then, they were separated into two groups. One group was grown in normal condition by flooding with water, while the other was flooded with 75 mM NaCl. After 21 days, the plants were collected and recorded as day 0 plants under normal condition, and the samples that were under the stress condition were also collected, and two days later they were treated with 75 mM of NaCl for RNA analysis, and were recorded as day 2. For the flower stage of the experiment, the rice plants were grown in soil in a 25 cm diameter pot, with one plant per pot. In the booting stage, for the salt stress treatment, 75 mM of NaCl was flooded over the soil, while the plants flooded with water were used as the controls. After three days, flag leaves were collected for RNA extraction, from the day 0 plants and the day 2 plants, after being exposed to the treatment. Table 1 displays the number of samples and conditions used in this study.

### 2.2. RNA Extraction and Sequencing

The total RNA was extracted from the plant tissues using the PureLink™ Plant RNA Reagent (Invitrogen, Carlsbad, CA, USA). The RNA-seq libraries were prepared according to the study of Udomchalothorn et al., [34]. The contaminated genomic DNA was treated with DNaseI (NEB), and the complementary DNA (cDNA) libraries were prepared using the KAPA stranded RNA-seq library preparation kit for Illumina^®^ (Kapa Biosystem, Davis, CA, USA). Fragments of 300 bp in size were chosen and connected with the adaptors. Then, all of the fragments were enriched using PCR for 12 cycles. All of the cDNA libraries of each treatment were sequenced using the Illumina HiSeq^TM^ 3000 sequencing system (Illumina, Davis, CA, USA). Three replicates of the RNA-seq libraries were used for the analysis. The complete genomic sequence of rice (*Oryza sativa* L.) was used as a reference [35]. The RNA-seq data consisted of 50 bp single-end reads (SRA BioProject ID: PRJNA507040). On average, there were approximately 20,665,662 reads per sample for the seedling stage and about 28,691,447 reads per sample for the booting stage. The quality control was performed according to the program of Pipeline of Parentally-Biased Expression (POPE) [36].

For the transcriptome data analysis, the adaptors of all of the short-sequence reads from the sequencing were removed, and then the sequence reads were categorized using the pipeline developed by Missirian et al. (2011) [37]. The sequence reads were aligned and mapped to the rice genome database (RGAP 6.0) (http://rice.plantbiology.msu.edu/, [35]) using Bowtie 2 [38] and TopHat [39]. The differentially expressed genes were determined using the *DESeq* package (version 1.24.0) [40].

### 2.3. Construction of a Gene Go-Expression Network

In order to construct a gene co-expression network, the RNA-seq read counts were analyzed and normalized using R package *DESeq* [40]. Non-informative genes, which were the genes whose RNA-seq read counts were zero for all of its replicates, were removed from the analysis. The gene co-expression networks were constructed for normal and salt-stress conditions, namely, normal-state and salinity-state networks, respectively. The gene expression levels of both the seedling and booting stages were combined for each condition. For the whole-state networks, all of the expression levels for the normal and salinity conditions were mixed together. Then nodes represent the genes and the edges represent the level of co-expression for any of the pairs of genes, which were the correlation values of the Pearson correlation coefficient of the expression levels. This resulted in a complete graph with edge weights of the correlation scores that were either positive or negative. When only high co-expression levels for constructing a binary network were concerned, the connections of any two genes existed when their absolute correlation coefficients were greater than or equal to 0.9. Finally, only the connected nodes were used to build up the networks.

### 2.4. Local Node and Network Properties

#### 2.4.1. Degree and Degree Assortativity

Degree is a commonly used measure for calculating the connectivity of a gene in a network. A high value degree can interpret how important an interesting gene is in the connection of a network [13]. Assuming that a gene node, *i*, is in a binary network, its’ degree can be calculated as the number of edges connected to it. For a weighted network, all of the connections in the network have their own values, rather than 0 or 1 as with a binary network. Therefore, a weighted degree was introduced as the sum of all of the weights of the edges connected to node *i*.

The Degree Assortativity is the Pearson correlation coefficient of the degree between pairs of connected nodes in a network [41]. It quantifies the tendency of the nodes being connected to other nodes that have similar degree or not. Positive values of this correlation mean any two connected nodes possessing a similar degree, while a negative correlation is represented by a network containing most of connected nodes with different degrees. All of the possible values of this correlation lie between −1 and 1, indicating the level of assortative patterns. When the correlation is 1, the network is said to be perfectly assortative, but when the correlation is −1, the network is completely disassortative. At the correlation of zero, the network is said to be non-assortative.

#### 2.4.2. Diameter Length

Diameter is defined as the maximal shortest path of all of the shortest paths between any two nodes of a network, which is the largest number of nodes to be traversed in order to travel from one node to another. For any disconnected graph, the diameter is defined as infinite [42].

#### 2.4.3. Clustering Coefficient

The clustering coefficient is a measure of the proportion of true connections and the number of all possible connections among the neighbors of a gene node. Assume that a gene node, i is an observed node while node j and node q are its neighbors. If there is connection between j and q, the connection between node i, j, and q occurs. According to Watts and Strogatz [43], the definition of the original clustering coefficient for a binary network is as follows.

(1) $$C\left(i\right)=\frac{\sum_{j}\sum_{q\neq j}\left(a_{\left(ij\right)}a_{\left(iq\right)}a_{\left(jq\right)}\right)}{k_{i}\left(k_{i}-1\right)},$$

where $a_{ij}$ is a binary value indicating the connection between node i and node j. $k_{i}$ is degree of node i. C(i) has value in range [0,1]. It is 0 if the neighbors of i do not connect to each other and it is 1 if all neighbor are pairwise connecting to each other. Based on a real-world network, mostly their nodes are connected with some level of strength connections or weights. Onnela et al. [27,28] was introduced the clustering coefficient for a weighted graph by taking a geometric mean of total weights of the neighbors as follows.

(2) $$C_{Onnela}\left(i\right)=\frac{\sum_{j}\sum_{q\neq j}{\left|w_{\left(ij\right)}w_{\left(iq\right)}w_{\left(ijq\right)}\right|}^{\frac{1}{3}}}{k_{i}\left(k_{i}-1\right)},$$

where $w_{ij}$ is the weight of the edge connecting node of i and j. These weights can be positive or negative. The value of $C_{Onnela}\left(i\right)$ is in the range [0,1] where 0 means there are no neighbors of i connected to each other, and 1 means that all of the neighbors are connected pairwise to each other with the highest weight. This formula uses the real weights in the network to compute both the numerator and denominator, while the original formula uses a cut-off to approximate the weights into a binary class, either connected or not connected.

## 3. Results

### 3.1. Overview of the Workflow

**Figure 1:**
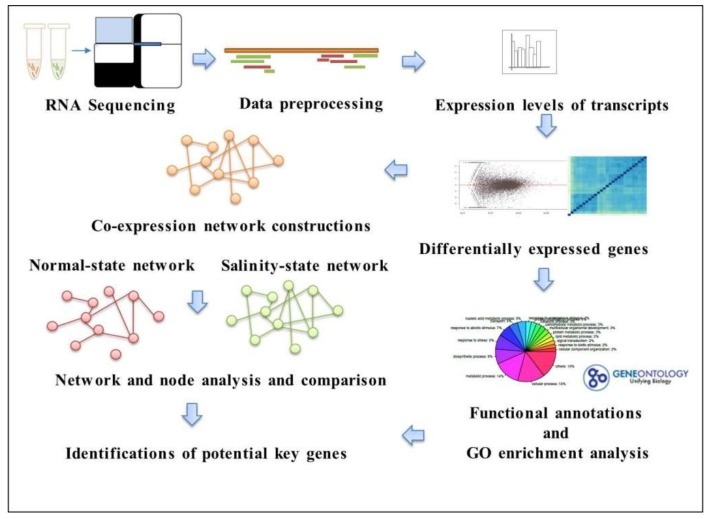
The analysis workflow to identify key genes with different state networks.

In order to identify the genes responding to salt tolerance, we considered two different states of a co-expression network. An overview of the workflow is shown in Figure 1. Firstly, all of the transcripts of genes under the salinity and normal conditions for the seedling and booting stages were sequenced using RNA-seq technology. The raw data were manipulated in the data preprocessing so as to retrieve the expression levels (read counts) for each transcript and then for the differential expression analysis. Later, the functional and gene ontology (GO) annotations were performed for all of the differentially expressed genes; either during the booting or seedling stages, and a GO enrichment analysis was performed. Then, the co-expression networks for salinity and normal conditions were constructed, and their network structures and properties were compared. The analysis results of the local network connections, such as the degree and clustering coefficient, were used to identify the genes related to salt tolerance.

### 3.2. Identify Differentially Expressed Genes Sensitive to Salinity in Rice

**Figure 2:**
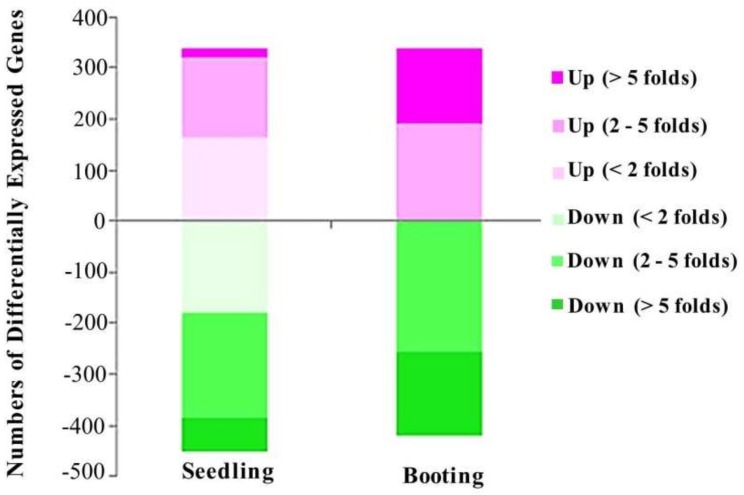
Fold change plot. Number of differentially expressed genes caused by salt stress treatment at the seedling stage and booting stage.

The rice stain KDML105, in the seedling and booting stages, was observed under salt-stress and control conditions, with three replicates each. The high-throughput transcriptome analysis of control and salt stress-treated rice samples was conducted using an Illumina HiSeq^TM^ 3000. We used the complete genomic sequence of rice (*O. sativa* L.) as a reference [35]. The comparison of the transcriptome differences of the reads between the control and the treated group was performed using *DESeq* package (version 1.24.0) [40] in R-programming language. The *p*-value was adjusted using Benjamini–Hochberg method for multiple-test correction. Finally, we obtained a total of 788 differentially expressed genes during the seedling stage, and 759 differentially expressed genes during the booting stage (with the adjusted *p*-value of less than 0.01), as described in Table 2. In total, there were 1472 differentially expressed genes (713 genes during the seedling stage only, 684 genes during the booting stage only, and 75 genes in both stages). In the seedling stage, salt stress caused the significant changes of 788 genes (Supplementary Table S1), while in the booting stage, 759 genes were found to have the differentially expression level due to salt stress (Supplementary Table S2). Among the differentially expressed genes, more than 50% of them were down-regulated by salt stress. Although the number of differentially expressed genes at the booting stage was lower than the number of differentially expressed genes at the seedling stage, stronger effects of the salt stress were found in the booting stage changing the level of all of the affected genes by more than two-fold (Figure 2).

### 3.3. Functional Annotations of Differentially Expressed Genes

**Figure 3:**
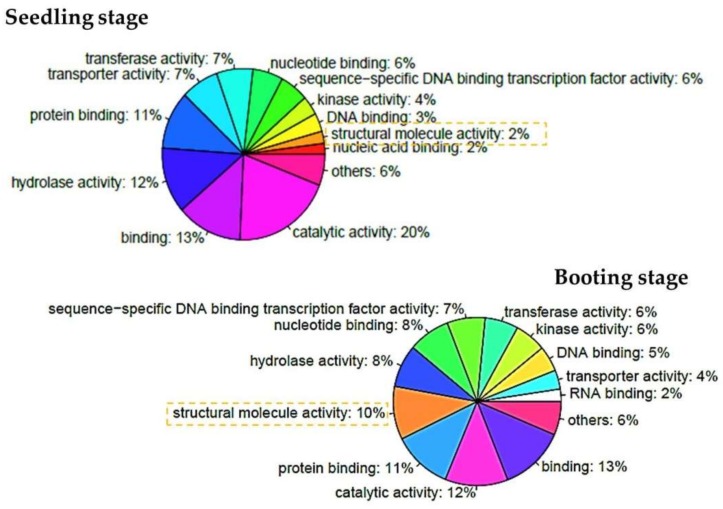
Gene ontology classification of differentially expressed genes among the molecular functions. The top-left of the pie chart represents the gene proportion of each function during the seedling stage. The bottom-right of the pie chart represents the gene proportion of each function during the booting stage.

To classify the function of the differentially expressed genes affected by salinity, the rice information and GO annotations for each gene locus were retrieved from http://rice.plantbiology.msu.edu/ [35]. In total, we annotated the GO terms for 718 differentially expressed genes during the seedling stage, and 666 genes during the booting stage (Supplementary Tables S3 and S4). The proportions of GO terms for both stages were similar (Supplementary Figure S1). Some differences were of interest and were discussed. The large proportions belonged to the genes that encoded the proteins with catalytic activity and binding in the molecular functions. The genes that encoded the proteins with hydrolase activity accounted for the next largest proportion during the seedling stage, but the proteins with a structural molecule activity were found more during the booting stage (Figure 3). Notice that during the booting stage, the differentially expressed genes encoded for proteins in the structural molecule activity were found more than these genes during the seedling stage. In both stages, the encoded proteins of the differentially expressed genes distributed through many cellular organelles. Furthermore, among the biological processes, many of these genes participated in cellular processes, metabolic processes, biosynthesis and the process of response to stress as expected. In order to better gain the enhanced functions of the differentially expressed genes, a GO enrichment analysis was performed using the Fisher’s exact test under a R programming environment. The GO enrichment showed that the differentially expressed genes of the seedling stage and the booting stage accounted for different functions as shown in Table 3. Obviously, the genes responding to the abiotic stimulus and stress were enriched during the seedling stage, and the genes related to the catalytic activity and the genes whose encoded proteins were localized in the extracellular region and cell wall were found most significantly during the seedling stage. During the booting stage, the genes whose encoded proteins were localized in the ribosome, nucleolus, cytosol, and vacuole, were found most when compared to the other gene ontology terms. Proteins with a structural molecule activity were enriched in this period as well. Furthermore, the genes responding to the translation process and the abiotic stimulus were found to be enriched during this period. Notice that genes responding to stress were found during the booting stage, but it is not significant after adjusting the *p*-value with multiple test correction when comparing with the other terms (as shown in Table 3 with the adjusted *p*-value by Benjamini–Hochberg correction for multiple testing). Since during the seedling stage, leaves are developing and grow, the process of metabolism is important; especially, carbohydrate metabolic process. The booting stage is the first step to develop rice into a reproductive phase in which rice need to develop seeds and flowers. Genes whose encoded proteins located in nucleolus, ribosome, vacuole and cytosol were found most and related to the translation of these genes.

### 3.4. Analysis of Co-Expression Networks in the Comparison of Salinity and Normal States of Rice

**Figure 4:**
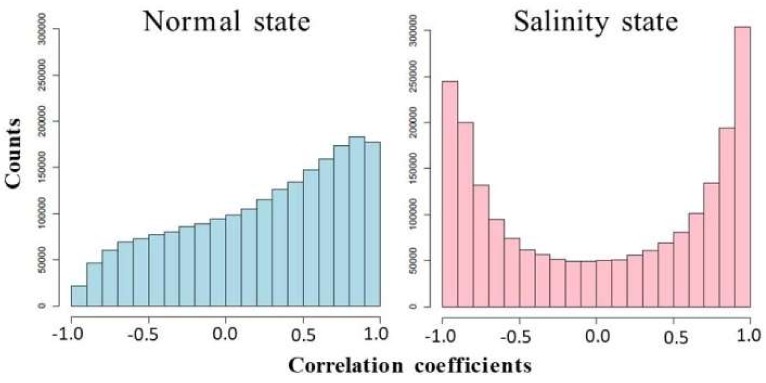
Distributions of correlation coefficients between the gene expression levels under normal and salinity states.

**Figure 5:**
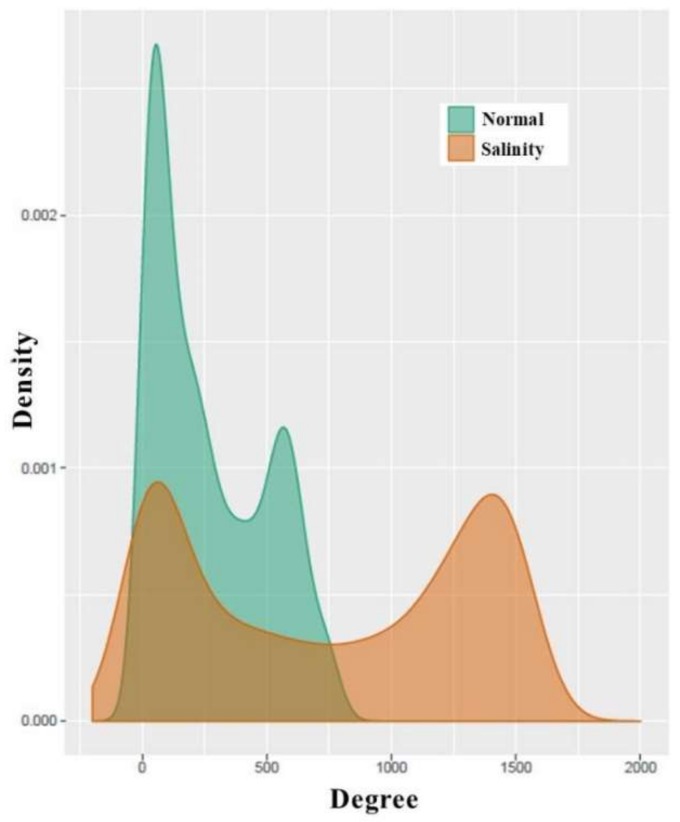
Degree distributions of normal-state and salinity-state networks.

**Figure 6:**
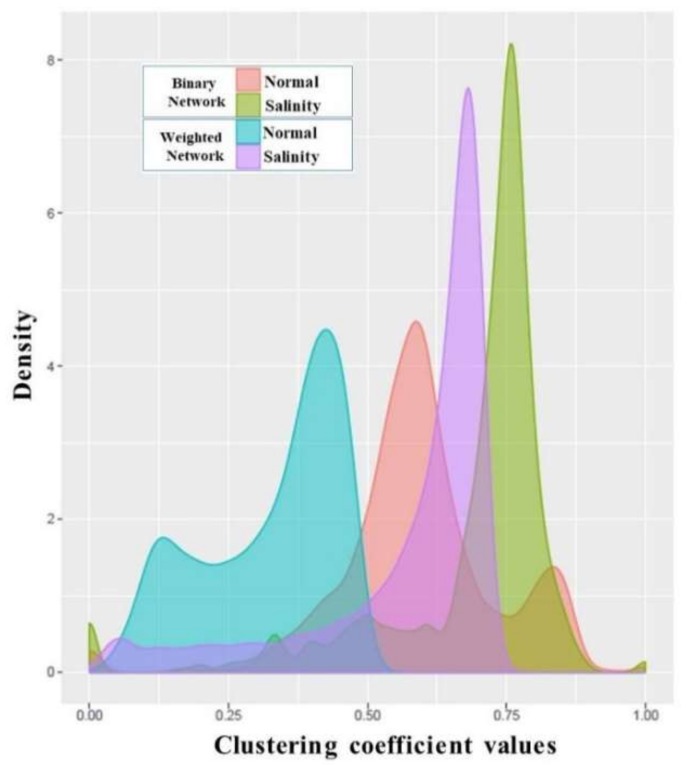
Clustering coefficient distributions of normal-state and salinity-state networks. Pink and green plots are the clustering coefficient distributions of the binary networks for normal and salinity states, respectively. The blue and purple plots are the clustering coefficient distributions of the weighted signed networks for the normal and salinity states.

To better understand how genes work together in a rice cell under different conditions, co-expression networks were built separately for the salinity and normal states. The expression levels of both the seedling and booting stages were used to calculate the correlations between the gene pairs of all of the differentially expressed genes. Interestingly, as shown in Figure 4, these genes under a salinity condition were more cooperated to each other than the genes under a normal situation, both in terms of negative and positive correlations. Notice that the negative correlations were found more under the salinity condition. This signified that these genes’ encoded proteins might have an “on” switch during the seedling stage, but “off” switch during the booting stage, or the other way round. Then, the binary co-expression networks for the salinity state and normal state were constructed by inferring network edges based on the high absolute correlation (>0.9). An analysis of the node properties for the different network structures of the salinity-state network and the normal-state network is shown in Table 4 and Figure 5 and Figure 6. In total, there were 1472 differentially expressed genes (713 genes during the seedling stage only, 684 genes during the booting stage only, and 75 genes were found in both stages). The salinity-state network contains 1446 genes connected to each other while the normal-state network contains 1443 genes connected to each other. Notice that in Table 4, the salinity-state network had more connections and was denser than the normal-state network as shown in their connections per node, average degree, number of hub and end nodes, and their diameter length. The Degree Assortativity showed that under both stages, these genes had similar degrees but were found much more in the normal situation than in the salinity. Global cluster densities of both networks were similar, with a bit more density in the salinity-state network. Many genes with a low degree in the normal-state network behaved differently and had more partners (or high degree) in the salinity state, as seen in Figure 5.

In general, the degree distribution of many real-world networks, including the co-expression network, follows the power-law distribution. Interestingly, in Figure 5, the degree distributions of both the normal and salt-stress networks follows a bimodal distribution, especially, in the salt-stress network. However, the normal network trended to follow the power-law distribution, as the majority of nodes had low degrees, even if the number of nodes with high degrees was more than the number of nodes expected by the power law. The salt-stress network clearly showed the bimodal distribution. This showed that a greater number of genes tended to have more co-partners during the salt stress.

To analyze how well each gene connected to its partners, rather than the utilization of the degree, examining the clustering coefficient is an alternative way. The distributions of the binary clustering coefficients (pink and green plots in Figure 6 for normal and salinity states, respectively) showed that most of the genes came to form a dense local cluster in the salinity condition rather than in the normal situation. The same tendency was also found when applying the weighted clustering coefficients (blue and purple plots in Figure 6 for the normal and salinity states, respectively). This means that under the normal state, most of the genes connected to each other were quite dense and had nearly the same local structure (similar clustering coefficient values), while under the salinity state, some genes behaved differently, and most turned out to have more connections among their partners. As expected, under the salt-stress condition, most of the genes behaved differently and needed to express more in order to release the stress, as well as to return to normal as fast as possible. The weighted clustering coefficients were smaller than the binary clustering coefficients as the network edges had weights ranging from −1 to 1 (the correlation values as shown in Figure 4). The negative weights had a direct effect on the clustering values.

### 3.5. Candidate Genes Responding to the Salinity Are Less Cooperative in the Normal

As shown in Table 4 and Figure 5, under salt-stress conditions more genes were co-expressed as many genes having more degrees. It is quite interesting that a certain gene with small partners in a normal situation gains more partners when it faces the stress. Therefore, our first list of candidate genes comprised genes showing small partners in a normal situation, but showing much higher partners in the salinity conditions. Initially, 484 genes were identified as small-degree genes (with degree < 50) under the normal situation. Then, the list was filtered to select the high hub genes (degree > 700) in the salinity-state network. Finally, the 135 hub genes and their corresponding degrees, clustering coefficients, and functional annotations are presented in Supplementary Table S5. This candidate list was enriched with the functions of the response to abiotic stimulus (GO:0009628) and the generation of precursor metabolites and energy (GO:0006091).

Of these genes, seventeen genes were found to be significantly differentially expressed genes under transcription levels that might directly affect the whole stress-release process (all of the list with their associated GO terms can be found in Table 5). Fifteen genes were found to have differential expressions in the booting stage, while only two genes were found in the seedling stage.LOC_Os05g45810, LOC_Os02g06330, LOC_Os09g29130, LOC_Os07g47140, LOC_Os05g37690, LOC_Os04g32460, LOC_Os05g02500, LOC_Os11g44810, and LOC_Os08g07970 were found to be down regulated in the booting stage. LOC_Os02g38040, LOC_Os04g32590, LOC_Os10g31850, LOC_Os12g06340, LOC_Os01g39770, and LOC_Os08g35190 were found to be up-regulated in booting stage. LOC_Os11g47920 and LOC_Os06g14750 were found to be upregulated in the seedling stage.

With the literature search, the candidates in Table 5 were grouped according to the evidence, if there was high evidence showing the function of salt stress in the plant, it is indicated as *** (>10 pieces of literature); ** for moderate evidence (from 5 to 10 pieces of literature); and * for some evidence (from 1 to 5 pieces of literature). Lastly, “-” means that is for no present evidence.

*** LOC_Os01g39770 (calcineurin B; CBL9), LOC_Os02g06330 (*AP2* domain containing protein), LOC_Os02g38040 (leucine-rich repeat family protein), LOC_Os05g45810 (calcineurin B; CBL4), LOC_Os08g07970 (OsbZIP64), and LOC_Os10g31850 (RING finger and *CHY* zinc finger domain-containing protein 1) were found to be related to performing the function of responding to salt, in various plants such as peanut, wheat, and *Arobidopsis thaliana*. For example, recently, *CBL4* and *CBL9* were found to be related to drought stress in wheat [44]. Interestingly, LOC_Os02g06330 (AP2 domain containing protein) was found in the transcriptional regulation under salinity stress in African rice [63].

** LOC_Os04g32460 (OsFBL16), LOC_Os05g37690 (OsFBL23) and LOC_Os07g47140 (CCT/B-box zinc finger protein) were found to be related to salt and drought stress in apple, soybean and Arabidopsis. OsFBL16 and OsFBL23 encoded for F-Box proteins, which play an important role in controlling the degradation of unneeded proteins during the cell cycle [134].

* LOC_Os05g02500 (OsMKP1, *GSN1*, dual specificity protein phosphatase) was studied in rice to regulate the activities of stress-related mitogen-activated protein kinases [91]. LOC_Os06g14750 (phosphatidylinositol-4-phosphate 5-Kinase family protein) and LOC_Os11g47920 (SCARECROW) were analyzed as salt-inducible genes in barley roots [93]. LOC_Os09g29130 (ZF-HD protein dimerization region containing protein) is a zinc-finger transcription factor that was studied recently as a novel regulator of the stress responsive gene. LOC_Os12g06340 (OsBLH1, BEL1-like homeodomain transcription factor) was studied in rice for the floret meristem [133].

- Currently, there is no study found for transcription factor LOC_Os04g32590 and auxin-repressed proteins LOC_Os08g35190 and LOC_Os11g44810.

**Figure 7:**
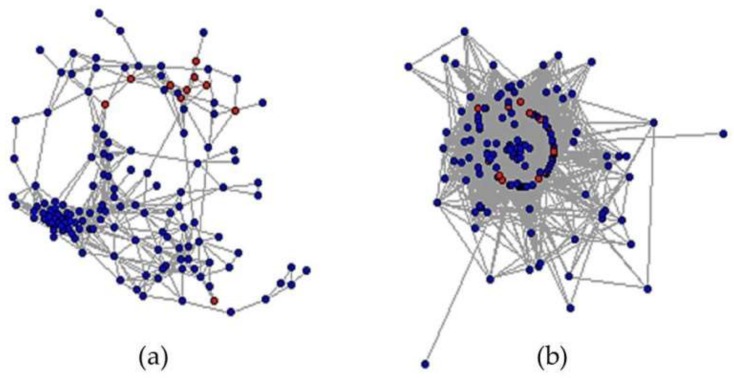
Co-expression networks under different states. Red nodes represent all seventeen candidates while blue nodes represent all of the other nodes. (**a**) Co-expression network of genes under normal situation. (**b**) Co-expression network of genes under salt stress.

The constructed co-expression network is shown in Figure 7. Due to the large and dense network, approximately 10% of all nodes plus 17 candidates were selected to plot the networks for each condition. Each node in the figure represents a differentially expressed gene, and each edge represents a connection between the two differentially expressed genes under different conditions. Notice that all seventeen candidates (red nodes in Figure 7) dispersed in the normal-state network while there were more connections in the salinity-state network.

## 4. Discussion and Conclusions

With the importance of Thai rice and the serious problem of saline soil in the northeastern part of Thailand, we proposed the two-state co-expression networks under normally grown and salt-stress conditions, in order to identify the key KDML105 gene markers responding to the salt stress. These two co-expression networks were constructed based on the gene expression levels investigated by RNA-seq technology during the seedling and booting stages, and then these data were compared in terms of the node and network properties. The results showed that the structure of the salinity-state network was denser than that of the normal-state network. Especially, when we applied the weighted clustering coefficient to the signed co-expression networks, more genes operated more partners in responding to the stress. This is clearly because under high salinity conditions there are more connections compared to the normal state. These changes in the gene regulation reveal that KDML105 rice under salt stress could be damaged, as salt or NaCl could stimulate a large change in cooperation among genes. This would help us to find a better list of genes whose encoded proteins were activated to interact and function more under salinity.

To select a potential list of genes for responding to the salt, the genes with few partners under normal state, but with many more partners under the salinity state were selected as the candidate key genes. Seventeen genes out of these genes were found in the transcription levels, and were validated with the literature search. Notice that out of these genes, *OsFBL23* and *OsFBL16* (LOC_Os05g37690 and LOC_Os04g32460, respectively), encoded for F-box proteins, were found down regulated during the booting stage. According to the analysis of their degrees and weighted clustering coefficients on the salinity-state network, they demonstrated a regulating role to control many other partners whose expression levels were correlated. F-Box proteins were found to be important for controlling the degradation of cellular proteins when they formed the Skp1p-cullin-F-box complex [134]. This protein complex plays an important role for unneeded protein degradation during the cell cycle. It has been found that several publications have reported that these proteins respond to salt stress [71,84,85,86,87,88,89]. Thus, it is crucial to investigate this degradation process as a pathway system in which OsFBL23 and OsFBL16 are involved.

Interestingly, the genes encoded for calcineurin B-like proteins, LOC_Os01g39770 (*CBL9*) and LOC_Os05g45810 (*CBL4*) were regulated differently. *CBL9* was upregulated while *CBL4* was downregulated during the booting stage. Both calcineurin genes were found in fourteen publications, which recently supported that they responded salt stress [44,45,46,47,48,49,50,51,52,53,54,55,56,57]. *CBL4* has been found to be acting as a calcium sensor involved in the regulatory pathway for the control of the intracellular Na^+^ and K^+^ homeostasis and salt tolerance [135]. For further experimental investigation and modeling, it would be interesting to focus on the effect of salinity in KDML105 rice when *CBL9* on chromosome 1 was switched on while *CBL4* on chromosome 5 was switched off during the salt stress.

In terms of the computational analysis, we found that the use of a network analysis is still a great tool to filter and select key genes under a specific condition. The construction of a co-expression network is the first and most important task of concern. In this work, the co-expression networks were built from the calculation of the Pearson correlation on the RNA-seq data. Small replicates and features directly affected the correlation values which also affected the network structure. It has been known that rice is sensitive to salt stress during the seedling and booting stages. Therefore, this work used the transcriptomic data of both the seedling and booting stages of KDML105 rice to construct the networks under normal-grown and salinity states. To distinguish the different structures of both networks, we found that the utility of the weighted networks revealed more curated structures and the change before and after the KDML105 rice was affected by the stress. In addition, the selection of the best key genes was performed with the use of degree because it obviously established the number of co-partners, while the clustering coefficient in this work showed a more or less similar local density. In principle, the framework of the analysis in this work can be applied to many other applications that are not only for KDML105 rice, but also for other plants and organisms as well as any other conditions. In addition, the development of the network analysis tool has become an important task to crop a large amount of genomic and transcriptomic data.

All in all, gaining more information in order to deal with the gene co-expression network at different stages can provide a better understanding of the complex regulatory systems responding to the environment, and also better candidates for further analysis and investigation.

## Figures and Tables

**Table 1:** Number of samples and conditions.

Summary	Detail
12 samples	three samples for each of condition and stage
2 conditions	Normal and salt stress
2 stages	Seedling (fully expanded leave) and booting (flag leave)
3 replicates	for each of condition and stage

**Table 2:** Number of differentially expressed genes under salt stress for the seedling and booting stages.

Stages	Gene Counts		Total
Seedling stage	Upregulated	337	788
	Downregulated	451	
Booting stage	Upregulated	338	759
	Downregulated	421	

**Table 3:** Gene ontology (GO) enrichment analysis for differentially expressed genes.

**Seedling Stage**					
**GO**	**Name**	**Type**	***p*-Value**	**Odds Ratio**	**Adjusted *p*-Value**
GO:0005576	extracellular region	CC	2.957 × 10^−9^	3.30	2.839 × 10^−7^
GO:0009628	response to abiotic stimulus	BP	1.305 × 10^−8^	1.79	1.24 × 10^−6^
GO:0005618	cell wall	CC	7.548 × 10^−7^	2.37	7.095 × 10^−5^
GO:0006950	response to stress	BP	2.539 × 10^−6^	1.54	0.000236
GO:0003824	catalytic activity	MF	0.000106	1.39	0.009719
GO:0005975	carbohydrate metabolic process	BP	0.000367	1.73	0.033430
GO:0008152	metabolic process	BP	0.000848	1.24	0.076302
GO:0009536	Plastid	CC	0.004486	1.24	0.399220
GO:0006091	generation of precursor metabolites and energy	BP	0.005245	1.88	0.461591
GO:0009579	Thylakoid	CC	0.013869	1.51	1
GO:0006629	lipid metabolic process	BP	0.015656	1.46	1
GO:0016020	Membrane	CC	0.017398	1.21	1
GO:0009987	cellular process	BP	0.018653	1.15	1
GO:0030312	external encapsulating structure	CC	0.035846	11.24	1
GO:0015979	Photosynthesis	BP	0.040445	1.70	1
GO:0040007	Growth	BP	0.040712	2.11	1
GO:0016049	cell growth	BP	0.046445	1.63	1
**Booting Stage**					
**GO**	**Name**	**Type**	***p*-Value**	**Odds Ratio**	**Adjusted *p*-Value**
GO:0005840	Ribosome	CC	2.91 × 10^−32^	6.27	2.80 × 10^−30^
GO:0005198	structural molecule activity	MF	8.05 × 10^−29^	5.58	7.65 × 10^−27^
GO:0006412	Translation	BP	9.25 × 10^−21^	4.13	8.69 × 10^−19^
GO:0005730	Nucleolus	CC	4.53 × 10^−11^	3.98	4.21 × 10^−9^
GO:0005829	Cytosol	CC	1.71 × 10^−9^	1.87	1.57 × 10^−7^
GO:0009628	response to abiotic stimulus	BP	2.90 × 10^−9^	1.92	2.64 × 10^−7^
GO:0005773	Vacuole	CC	8.21 × 10^−6^	1.91	0.000739
GO:0005618	cell wall	CC	0.007243	1.61	0.644585
GO:0040007	Growth	BP	0.007826	2.90	0.688678
GO:0009606	Tropism	BP	0.013779	3.50	1
GO:0019748	secondary metabolic process	BP	0.022283	1.74	1
GO:0006950	response to stress	BP	0.027036	1.21	1

MF: Molecular Functions; CC: Cellular Components; BP: Biological Processes.

**Table 4:** Node and network properties.

Properties	Normal-State Network	Salinity-State Network
Number of nodes	1446	1443
Number of edges	98,754	273,620
Connections per node	68	190
Average degree	137	379
Number of hub nodes (degree > 200)	448	908
Number of end nodes (degree = 1)	11	7
Diameter length	11	10
Degree Assortativity	0.7100	0.3739
Global clustering coefficient	0.5924	0.6871

**Table 5:** List of candidate key genes responding to the salinity.

Locus_ID	Stage	up/down	Function	References	Mark
LOC_Os01g39770	booting	up	calcineurin B, putative, expressed	[44,45,46,47,48,49,50,51,52,53,54,55,56,57]	***
LOC_Os02g06330	booting	down	*AP2* domain containing protein, expressed	[58,59,60,61,62,63,64,65,66,67,68,69,70]	***
LOC_Os02g38040	booting	up	leucine-rich repeat family protein, putative, expressed	[71,72,73,74,75,76,77,78,79,80,81,82,83]	***
LOC_Os04g32460	booting	down	OsFBL16-F-box domain and *LRR* containing protein, expressed	[71,84,85,86,87,88,89]	***
LOC_Os04g32590	booting	up	transcription factor, putative, expressed	-	-
LOC_Os05g02500	booting	down	OsMKP1, *GSN1*, dual specificity protein phosphatase, putative, expressed. A calmodulin-binding mitogen-activated protein kinase phosphatase induced by wounding and regulating the activities of stress-related mitogen-activated protein kinases in rice	[90,91]	*
LOC_Os05g37690	booting	down	OsFBL23-F-box domain and *LRR* containing protein, expressed	[71,84,85,86,87,88,89]	***
LOC_Os05g45810	booting	down	calcineurin B, putative, expressed	[44,45,46,47,48,49,50,51,52,53,54,55,56,57]	***
LOC_Os06g14750	seedling	up	phosphatidylinositol-4-phosphate 5-Kinase family protein, putative, expressed	[92,93]	*
LOC_Os07g47140	booting	down	CCT/B-box zinc finger protein, putative, expressed	[94,95,96,97,98,99,100]	**
LOC_Os08g07970	booting	down	OsbZIP64 [101], transcription factor, putative, expressed	[102,103,104,105,106,107,108,109,110,111,112,113,114,115,116]	***
LOC_Os08g35190	booting	up	auxin-repressed protein, putative, expressed	-	-
LOC_Os09g29130	booting	down	ZF-HD protein dimerization region containing protein, expressed	[117,118]	*
LOC_Os10g31850	booting	up	RING finger and *CHY* zinc finger domain-containing protein 1, putative, expressed	[119,120,121,122,123,124,125,126,127,128,129,130,131,132]	***
LOC_Os11g44810	booting	down	auxin-repressed protein, putative, expressed	-	-
LOC_Os11g47920	seedling	up	SCARECROW, putative, expressed	[93]	*
LOC_Os12g06340	booting	up	OsBLH1, *BEL1*-like homeodomain transcription factor, putative, expressed	[133]	*

## Supplementary Materials

The following are available online at http://www.mdpi.com/2073-4425/9/12/594/s1, Figure S1: Gene ontology classification of differentially expressed genes, Table S1: Differentially expressed genes under seedling stage, Table S2: Differentially expressed genes under booting stage, Table S3: Gene ontology (GO) mapping for differentially expressed genes under seedling stage, Table S4: Gene ontology (GO) mapping for differentially expressed genes under booting stage, Table S5: Candidate key genes related with transcription level.
