# Supplementary material for: Two-State Co-Expression Network Analysis to Identify Genes Related to Salt Tolerance in Thai Rice
Source: Genes (Basel). 2018 Nov 29;9(12):594. doi: 10.3390/genes9120594 (PMC6316690; doi:10.3390/genes9120594)

Seedling stage

Molecular Functions

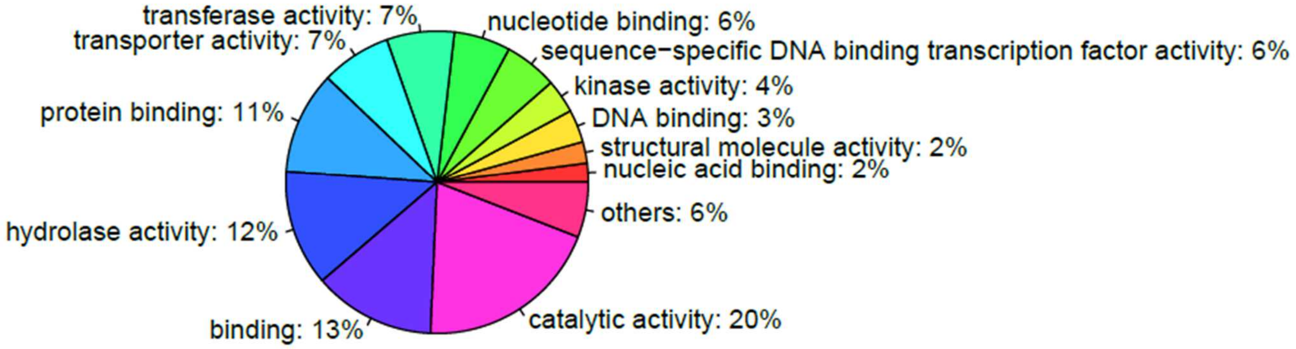

Cellular Components

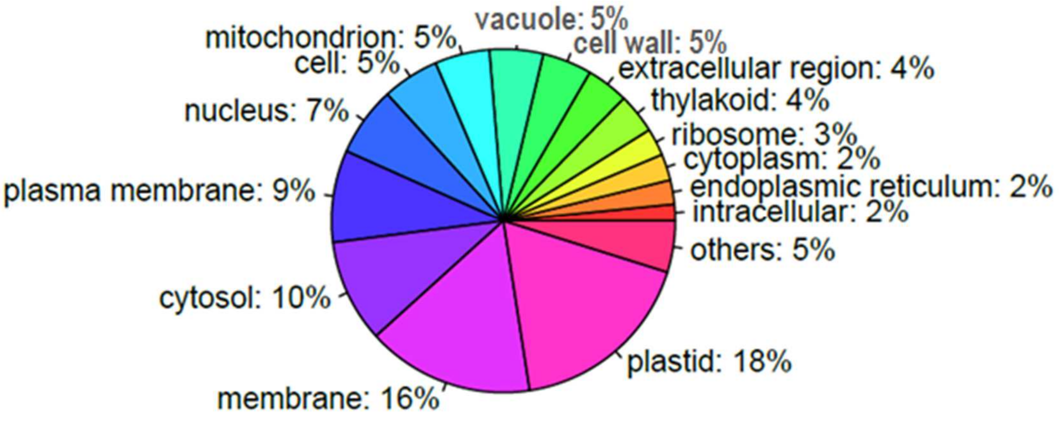

Biological Processes

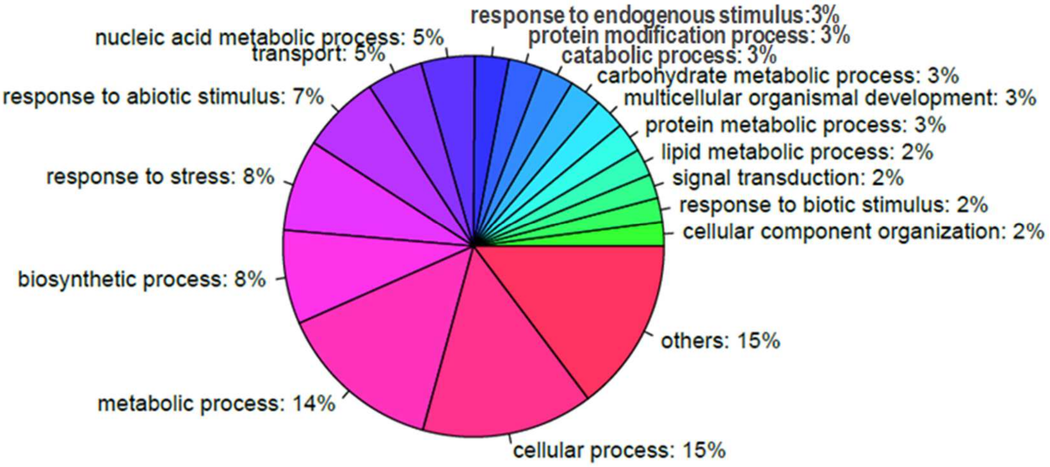

Booting stage

Molecular Functions

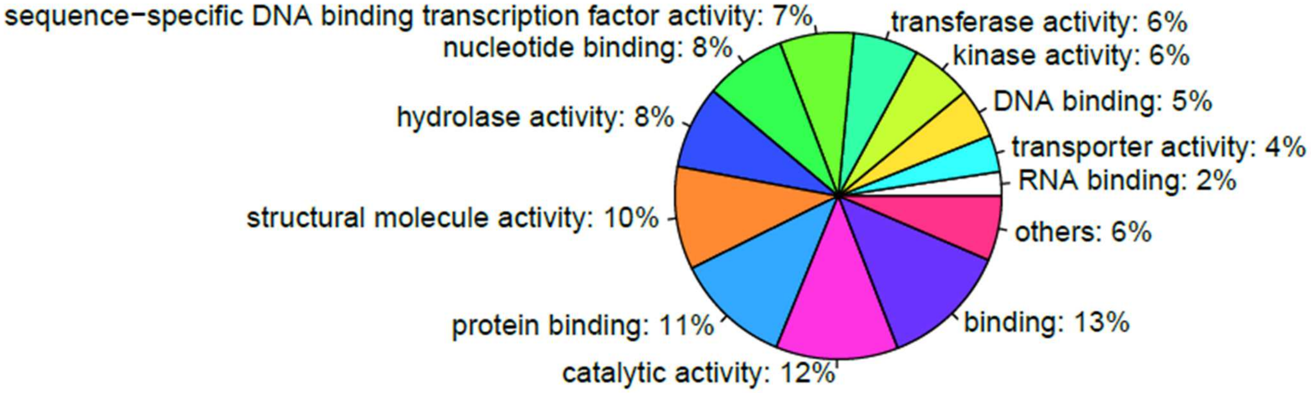

Cellular Components

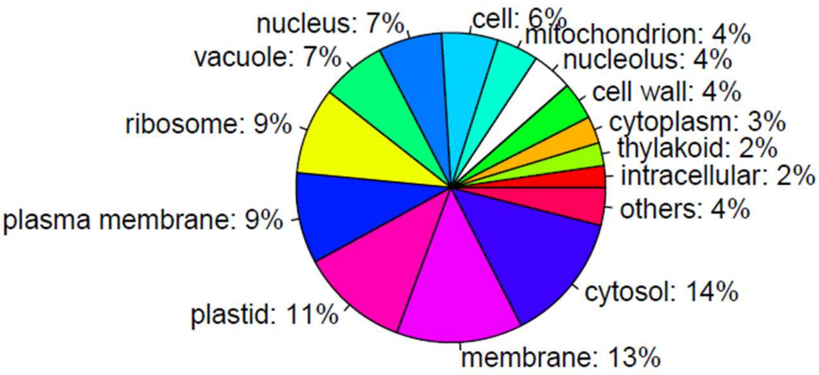

Biological Processes

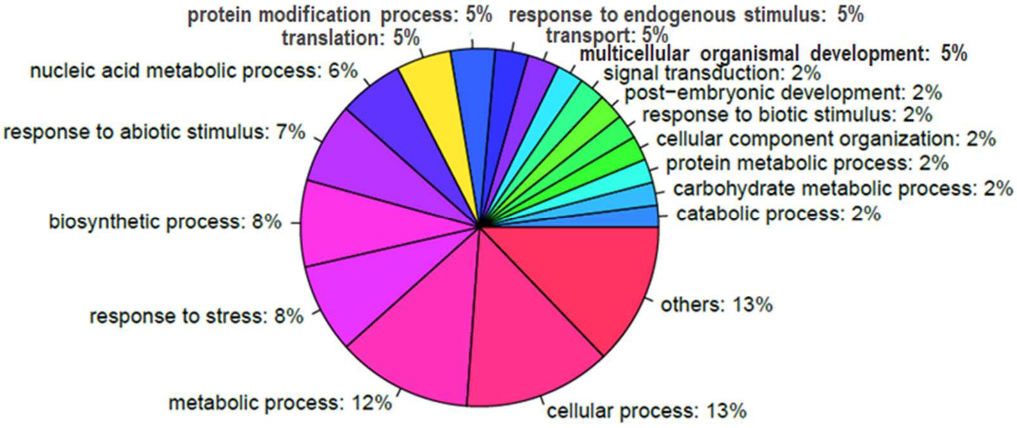

Supplement: Supplementary file 1 [file genes-09-00594-s001.zip › Supplement/FigureS1_GOclassification-08112018.pdf]
